# DNA/RNA transverse current sequencing: intrinsic structural noise from neighboring bases

**DOI:** 10.3389/fgene.2015.00213

**Published:** 2015-06-19

**Authors:** Jose R. Alvarez, Dmitry Skachkov, Steven E. Massey, Alan Kalitsov, Julian P. Velev

**Affiliations:** ^1^Department of Physics, University of Puerto RicoSan Juan, PR, USA; ^2^Department of Biology, University of Puerto RicoSan Juan, PR, USA; ^3^Department of Physics, University of NebraskaLincoln, NE, USA; ^4^Université Grenoble Alpes/CNRS/CEA, INAC-SPINTECGrenoble, France

**Keywords:** DNA, RNA, third-generation sequencing, nanopore sequencing, transverse current

## Abstract

Nanopore DNA sequencing via transverse current has emerged as a promising candidate for third-generation sequencing technology. It produces long read lengths which could alleviate problems with assembly errors inherent in current technologies. However, the high error rates of nanopore sequencing have to be addressed. A very important source of the error is the intrinsic noise in the current arising from carrier dispersion along the chain of the molecule, i.e., from the influence of neighboring bases. In this work we perform calculations of the transverse current within an effective multi-orbital tight-binding model derived from first-principles calculations of the DNA/RNA molecules, to study the effect of this structural noise on the error rates in DNA/RNA sequencing via transverse current in nanopores. We demonstrate that a statistical technique, utilizing not only the currents through the nucleotides but also the correlations in the currents, can in principle reduce the error rate below any desired precision.

## Introduction

Deoxyribonucleic acid (DNA) and ribonucleic acid (RNA) are two types of biological macromolecules essential for the functioning and reproduction of all living organisms. Both are heteropolymers of four nucleobases, adenine (*A*), guanine (*G*), cytosine (*C*), and thymine/uracil (*T*/*U*), where thymine is replaced by uracil (unmethylated form of thymine) in RNA. Because of its role in encoding the genetic information in humans and respectively its importance for medicine, DNA has been the object of an enormous effort to develop methods to routinely determine its sequence. The existing array of methods have been classified in three generations (Schadt et al., 2010). The first generation is based on the original Sanger sequencing method, which is an involved biochemical process of DNA fragmentation, amplification and chain termination, optical detection, and computer-based sequence assembly (Sanger and Nicklen, 1977). The second generation or next-generation sequencing (NGS) methods achieve larger throughput via DNA cloning and amplification and massively parallel processing (Shendure and Ji, 2008). Notwithstanding the vastly improved output and reduced cost of obtaining genome sequences, the technologies remain prone to errors such as amplification bias and misalignment of base repeats (Metzker, 2010). The cloning and amplification process limit the read length to a few hundred bases and high coverage is necessary for improved accuracy. An additional disadvantage is that base modifications, such as methylation (Korlach and Turner, 2012), are lost during the process. The third generation of sequencing techniques, exploits physical methods for DNA detection on the level of single-molecule detection (Bayley, 2006; Branton et al., 2008; Zwolak and Di Ventra, 2008). Among the variety of proposed techniques the nanopore sequencing has emerged as one of the most promising (Wang et al., 2015). By eliminating the error producing amplification step, long read lengths become possible which also greatly simplifies the assembly process. This technique promises to significantly extend read length while reducing the amount of starting material. Moreover, the nanopore sequencing technique is not limited to DNA, but it should be capable of sequencing RNA and other biological macromolecules.

Two main approaches to DNA/RNA nanopore sequencing are under development—ionic blockade current through the nanopore (Kasianowicz et al., 1996) and transverse tunneling current between electrodes on the nanopore (Zwolak and Di Ventra, 2005). The ionic current method takes advantage of the difference in the spatial extension of the nucleobases. As a single strand DNA or RNA molecule suspended in electrolyte solution is driven through a protein nanopore by electric field, the variations of the ionic current as the nucleotides block the pore channel can be correlated to the type of the nucleotide (Kasianowicz et al., 1996; Akeson et al., 1999; Clarke et al., 2009; Stoddart et al., 2009; Ayub and Bayley, 2012; Ayub et al., 2013; Cracknell et al., 2013). Solid-state analogs of protein nanopore offer better control of the dimensions and the properties of the channel (Li et al., 2001; Dekker, 2007). DNA detection in solid-state nanopore has also been demonstrated (Fologea et al., 2005; Storm et al., 2005; Gershow and Golovchenko, 2007; Wanunu et al., 2009). Achieving high fidelity, nevertheless, is difficult because the interaction of DNA with the solution, the DNA-nanopore interaction, and temperature-induced fluctuations affect the ion blockage current. This is currently an area of development where methods are continuously being developed to address these problems, such as immobilization of the base in the nanopore (Purnell et al., 2008), fitting adaptors in the pore (Clarke et al., 2009), or tagging the bases (Singer et al., 2010), as well as statistical processing to improve accuracy of base calling (Timp et al., 2012).

The transverse current method exploits the difference in electronic structure of the bases, rather than their geometry. In this case electrodes are deposited on the nanopore and the transverse electronic current through the nucleotides is measured as they translocate through the pore (Zwolak and Di Ventra, 2005; Lagerqvist et al., 2006; Di Ventra et al., 2012; Di Ventra, 2013). The tunneling current is sensitive to the electronic structure of the molecule and can be used to identify the base, which has been demonstrated by contacting a single nucleotide between an electrode and a scanning tunneling microscope tip (He et al., 2008; Shapir et al., 2008). Electrodes have also been manufactured on solid-state nanopore (Maleki et al., 2009; Tsutsui et al., 2011) and base identification by tunneling current has been demonstrated for both DNA and RNA (Tsutsui et al., 2010; Ivanov et al., 2011; Ohshiro et al., 2012). Dynamic or environmental disorder, coming from the interaction of the DNA/RNA with the environment at finite temperature, strongly modifies the tunneling current giving rise to a large current spread (Lagerqvist et al., 2007; Krems et al., 2009). Similarly to the ionic current case, the dynamic noise can be minimized by fitting the nanopore with adaptors (Chang et al., 2010). Moreover, multiple measurements of the same base can be used to sample the distribution of the values of electron current for each base, which are statistically distinguishable (Lagerqvist et al., 2006, 2007; Ohshiro et al., 2012).

The transverse current method, however, is prone to a different type of noise deriving from disorder along the chain of the DNA molecule itself. Most theoretical work has been concentrated on the study of dynamic noise and much less attention has been paid to this static noise. Earlier theoretical calculations suggested that this noise should be rather small (Zwolak and Di Ventra, 2005), however, the study involved only very short sequences (triplets) for small voltages and the data actually showed very significant changes of the current. Actual current reads of short DNA/RNA chains also clearly demonstrate that the current through the same bases, after the environmental noise is averaged out, differs depending on their environments (Ohshiro et al., 2012). Thus, the current spread is due to a large part to the structural noise arising from the randomness of the base sequence along the chain. In a recent work we performed a comprehensive theoretical study of effect of the structural noise on transverse current within a parametrized tight-binding (TB) model (Alvarez et al., 2014). It was shown that the static noise would exist even if the molecule is perfectly stationary with respect to the electrodes and that it could be comparable to that of the environmental noise, thus, introducing very large error rates. This noise is an electronic structure effect arising from modifications of the molecular orbitals (MOs) of each base due to interaction with the MOs of its neighbors (Miroshnichenko et al., 2010). Statistical proceeding accounting for correlations between the currents through neighboring bases could greatly increase the accuracy of base calling.

In this work we continue the investigation of the transverse current nanopore sequencing. Previously we used a parametrized single-level TB model for transport in DNA, which only accounts for the influence of the highest occupied molecular orbitals levels on the transport and the quality of the parameterization is difficult to assess. Here we use a multi-orbital TB Hamiltonian derived from first principles calculations, which accounts for the influence of a specified number of MOs around the bias window. This allows us to improve the precision of the predictions, but also to extend the applicability of the model beyond DNA. We demonstrate that nanopore sequencing works equally well for RNA, which opens a whole new area of biological applications.

## Methods

### Multi-orbital model for DNA/RNA chains

**Figure 1:**
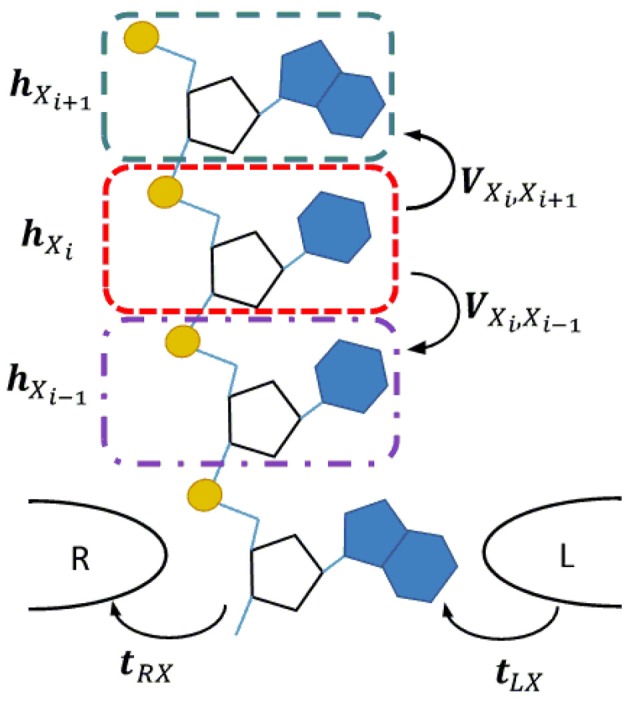
**Transverse current setup for a single strand DNA or RNA molecule translocating through a nanopore between a pair of tapered metal electrodes**. In the corresponding linear chain model each nucleotide is represented by a site (color dashed line rectangles). The quantities ***h***_*X*_*i*__ and ***V***_*X*_*i*_*X*_*j*__ represent the Hamiltonian of an isolated site and the interaction between neighboring sites. The coupling of a nucleotide with the right/left electrode are represented by ***t***_(*R*/*L*)*X*_.

The DNA/RNA nanopore sequencing geometry is schematically illustrated in Figure 1. We assume that a single-strand DNA/RNA molecule translocates between two tapered electrodes which make contact with one nucleotide at a time. To represent a polynucleotide we adopt the linear model for DNA transport, which represents the DNA molecule with one site per nucleotide (Apalkov et al., 2007; Macia, 2009). In this model each nucleotide is represented as a single site and the DNA/RNA heteropolymer as a chain of such sites. In order to improve the predictive power of the model, instead of a single energy level per site, we consider multiple energy levels corresponding to the MOs in the energy region around the bias window which is active in the carrier transport. Since we are interested only in the intrinsic or static source of noise, we consider low temperature in which case each site has a well specified position with respect to the electrodes.

DNA/RNA are large and complex molecules and *ab initio* approaches are the most appropriate tools for obtaining the exact electronic structure, however, full first-principles calculations of electronic structure of a polynucleotide molecule is still a very demanding task, especially in the case when long molecules and large statistics are required. For that reason our approach is to perform first-principles calculations of short DNA/RNA chains and extract from them a smaller rank Hamiltonian matrix describing correctly the MOs in the active energy window. Subsequently we use this Hamiltonian in the transport calculations.

#### DNA/RNA fragment electronic structure from first principles

The electronic structure of the DNA/RNA chains is calculated from first principles within the density functional theory as implemented in the Amsterdam Density Functional (ADF) package (Fonseca Guerra et al., 1998; Te Velde et al., 2001). We use the Perdew-Burke-Ernzerhof (PBE) exchange and correlation functional with triple-zeta polarized (TZP) basis set. Test calculations of DNA homopolymers show that interactions between fragments decay very rapidly with the distance, such that the second nearest-neighbor interaction is two orders of magnitude smaller than that between first nearest-neighbors. Thus, it is an excellent approximation to adopt a TB representation with first nearest-neighbor interaction only. Within this approximation, the calculations of all individual DNA/RNA nucleotides, *X*, and all possible nucleotide pairs, *XY*, are sufficient to extract the onsite and the nearest-neighbor hopping Hamiltonian matrix elements. Here *X*, *Y* ∈ *A*, *G*, *C*, *T* in the case of DNA and *X*, *Y* ∈ *A*′, *G*′, *C*′, *U* in the case of RNA. The *A*, *G*, and *C* bases are identical in DNA and RNA, however, the prime signifies the different backbone in the case of RNA, *U* in RNA is an unmethylated form of the DNA base *T*.

In order to insure that the basis is identical for the same nucleotide in all independent calculations, we use the fragment molecular orbital (FMO) theory (Kitaura et al., 1999). The molecule is split into fragments and the eigenvectors of these fragments are used, instead of atomic basis functions, to be the basis for the calculation of the total system. In the case of DNA/RNA each nucleotide (base + backbone) is chosen as a separate fragment, as shown in Figure 1. The calculation is performed in ADF in two steps. First, the electronic structure of each fragment *i* is solved separately and the MOs for each are found **Φ**_*i*_ = {ϕ_*i*α_} where α indices the fragment MOs. Second, the combined MOs of all fragments, **Φ** = {**Φ**_***i***_}, are taken as a new basis set in which the MOs of the entire system, **Ψ**, is expressed as a linear combination, **Ψ** = ***C*****Φ**. The matrix of expansion coefficients, ***C***, is obtained by solving the generalized eigenvalue equation ***HC*** = **ε** ***SC***, where ***H***_*i*α, *j*β_ = 〈 ϕ_*i*α_ | $\hat{H}$| ϕ_*j*β_ 〉 and ***S***_*i*α, *j*β_ = 〈 ϕ_*i*α_ | ϕ_*j*β_〉 are the Hamiltonian and overlap matrix elements between fragment orbitals ϕ_*i*α_ and ϕ_*j*β_, and **ε** is the diagonal matrix of the corresponding energy levels of the system. The quantities, ***H***, ***S***, ***C***, **ε**, are calculated within ADF from first principles. Here ***H*** and ***S*** are very large matrices containing all the MOs of the nucleotide.

#### Reduced fragment hamiltonian

The full Hamiltonian for a reasonably long DNA/RNA chain would be too large to perform transport calculations. However, we can take advantage of the fact that only the MOs in the active energy window participate in the transport. Truncating the Hamiltonian to only the *m* MOs with energies within the active window, however, is not trivial because these orbitals are hybridized with all other orbitals in the system. To account for this hybridization we project the full Hamiltonian on the desired levels using projector operator techniques (Kurnikov and Beratan, 1996; De Andrade and Freire, 2003, 2004; Soriano and Palacios, 2014). These techniques have been successfully applied to description of long-range electron transfer in proteins (Kurnikov and Beratan, 1996; De Andrade and Freire, 2004) and the conductance of metallic nanocontacts (Soriano and Palacios, 2014).

The MOs of the system {|Ψ_*i*_〉} belong to a Hilbert space defined by the non-orthogonal basis set {|ϕ_*i*_〉} of the MOs of the fragments. The metric of this space is defined by the overlap matrix elements, ***S***_*ij*_ = 〈 ϕ_*i*_| ϕ_*j*_〉. Assuming a complete basis, the closure relation can be written as $\hat{I}$ = ∑_*i*_$\hat{P}$_*i*_, where ${\hat{P}}_{i}=|\phi_{i}\rangle \sum_{j}{\left[S^{-1}\right]}_{ij}\langle \phi_{j}|$ is the projection operator on the state |ϕ_*i*_〉. The Hilbert space can be split into two non-orthogonal complementary subspaces *P* and *Q*, where *P* represents the active energy region and *Q* represents the rest of the system. Thus, the basis set is divided into two subsets, $\left\{|\phi_{p}\rangle \right\}\in P$, and $\left\{|\phi_{q}\rangle \right\}\in Q$, where the projector operators onto *P* and *Q* subspaces are defined as:

(1) $$\hat{P}=\sum_{i\in P}|\phi_{i}\rangle \sum_{j\in \left\{P,\;Q\right\}}{\left[S^{-1}\right]}_{ij}\langle \phi_{j}|,\hat{Q}=\sum_{i\in Q}|\phi_{i}\rangle \sum_{j\in \left\{P,\;Q\right\}}{\left[S^{-1}\right]}_{ij}\langle \phi_{j}|$$

where it can be shown that they obey the standard relations for projection operators $\hat{P}$$\hat{P}$ = $\hat{P}$, $\hat{Q}$$\hat{Q}$ = $\hat{Q}$, $\hat{P}$$\hat{Q}$ = $\hat{P}$$\hat{Q}$ = 0, and have the completeness property $\hat{P}$ + $\hat{Q}$ = $\hat{I}$. Since the subspaces are non-orthogonal the projection operators are non-Hermitian $\hat{P}$ ≠ $\hat{P}$^†^ and $\hat{Q}$ ≠ $\hat{Q}$^†^.

With the help of these operators we can project the Schrödinger equation of the system, (*E* − $\hat{H}$)|Ψ 〉 = 0, on the *P* and *Q* substpaces to obtain the system of equations:

(2) $$\begin{array}{l} {\hat{P}}^{†}\left(E-\hat{H}\right)\hat{P}\hat{P}|\Psi \rangle =-{\hat{P}}^{†}\left(E-\hat{H}\right)\hat{Q}\hat{Q}|\Psi \rangle \\ {\hat{Q}}^{†}\left(E-\hat{H}\right)\hat{P}\hat{P}|\Psi \rangle =-{\hat{Q}}^{†}\left(E-\hat{H}\right)\hat{Q}\hat{Q}|\Psi \rangle \end{array}$$

where we have used the idempotency and the completeness relation of the projection operators to arrive at this form. This system of equations can be solved for the part of the wavefunction projected on the active window, $\hat{P}$|Ψ 〉, as follows:

(3) $$\begin{array}{l} \left[{\hat{P}}^{†}\hat{H}\hat{P}+{\hat{P}}^{†}\left(E-\hat{H}\right)\hat{Q}{\hat{Q}}^{†}{\left(E-\hat{H}\right)}^{-1}\hat{Q}{\hat{Q}}^{†}\left(E-\hat{H}\right)\hat{P}\right]\hat{P}|\Psi \rangle \\ \;=E\hat{P}|\Psi \rangle \end{array}$$

which is the Schrödinger equation for an effective Hamiltonian ***H***_*eff*_(*E*) = ***H***_*PP*_ + (*E**S***_*PQ*_ − ***H***_*PQ*_)***G***_*QQ*_(*E**S***_*QP*_ − ***H***_*QP*_), where ***H***_*XY*_ = $\hat{X}$^†^ $\hat{H}$$\hat{Y}$ and ***S***_*XY*_ = $\hat{X}$^†^ $\hat{Y}$ with $\hat{X}$,$\hat{Y}$ ∈ {$\hat{P}$, $\hat{Q}$}. Furthermore, the quantity ***G***_*QQ*_ = (*E**S***_*QQ*_ − ***H***_*QQ*_)^−1^ is the projection of the Green's function (GF) operator on the *Q*. Alternatively the expression for the effective Hamiltonian can be derived from projection of the Dyson equation for the GF

(4) $$H_{eff}(E)=ES_{PP}+{\left[G_{PP}(E)\right]}^{-1}$$

where ***S***_*PP*_ and ***G***_*PP*_ are the overlap matrix and GF projected on the active region. We notice that the price of the Hamiltonian reduction is that it becomes explicitly energy dependent.

#### DNA/RNA chain construction from fragments

The Hamiltonian operator of a chain of nucleotides can be written as $\hat{\text{H}}=\sum_{i}{\hat{h}}_{i}+\sum_{i,j}{\hat{V}}_{ij}$ where $\hat{h}$_*i*_ is the Hamiltonian of an isolated fragment and $\hat{V}$_*ij*_ the interaction between fragments. The onsite matrix elements are $\varepsilon_{i\alpha ,i\beta }=\langle \phi_{i\alpha }\left|\hat{H}\right|\phi_{i\beta }\rangle$, and the hopping parameters $V_{i\alpha ,j\beta }=\langle \phi_{i\alpha }\left|\hat{H}\right|\phi_{j\beta }\rangle$ where we set to zero ***V***_*i*, *j*_ = 0 for all *j* ≠ *i* ± 1. Within the two-center approximation the onsite matrix elements contain contributions from the neighboring nucleotides $\varepsilon_{i\alpha ,i\beta }=\langle \phi_{\text{i}\alpha }\left|{\hat{h}}_{i}\right|\phi_{i\beta }\rangle +\langle \phi_{\text{i}\alpha }\left|{\hat{V}}_{i,i-1}\right|\phi_{i\beta }\rangle +\langle \phi_{\text{i}\alpha }\left|{\hat{V}}_{i,i+1}\right|\phi_{i\beta }\rangle$. By performing first-principles calculations of single nucleotides *X*_*i*_ and pairs of nucleotides *X*_*i*_*X*_*j*_ we can extract all onsite ***h***_*X*_*i*__ and interaction ***V***_*X*_*i*_*X*_*j*__ matrix elements and then project them on the same set of fragment MOs to produce the reduced Hamiltonians ***$\tilde{h}$***_*X*_*i*__ and ***$\tilde{V}$***_*X*_*i*_*X*_*j*__. Having these building blocks we can construct the Hamiltonian of an arbitrary DNA/RNA chain as a banded block matrix given by:

(5) $$\begin{array}{l} \varepsilon_{X_{i\alpha \beta }}={\tilde{h}}_{X_{i,\alpha \beta }}+{\tilde{V}}_{X_{i,\alpha }X_{i+1,\beta }}+{\tilde{V}}_{X_{i,\alpha }X_{i-1,\beta }},\\ \;V_{i\alpha ,(i\pm 1)\beta }={\tilde{V}}_{X_{i,\alpha }X_{i\;\pm \;1,\beta }} \end{array}$$

where the terms ${\tilde{V}}_{X_{i\alpha }X_{i\;+\;1,\beta }}$ and ${\tilde{V}}_{X_{i\alpha }X_{i-1,\beta }}$ introduce a renormalization factor on the onsite energy at site *i* coming from the neighbors nucleotides on sites *i* ± 1. This process produces a multi-orbital TB model which is derived directly from first-principles calculations without uncontrolled approximations. The size of the active region, i.e., number of valence MOs, is the main parameter the effect of which can easily be controlled by increasing the number of levels in the description.

A lot of the previous work on transport in DNA takes into account only the frontier orbitals, i.e., the highest occupied molecular orbital (HOMO) and lowest unoccupied molecular orbitals (LUMO), of the molecule within parametrized models (Iguchi, 2001; Roche, 2003; Shih et al., 2008; Hawke et al., 2010). However, the HOMO-LUMO description has been shown to be deficient in principle because the current is carried by many MOs which produce distinct features in the I–V curves (Heurich et al., 2002). Moreover, the validity of the parametrization is difficult to assess and a new parametrization is necessary for each new system. This approach improves considerably the description electronic properties beyond standard frontiers orbitals model (LUMO-HOMO) and opens the possibility for considering systems beyond DNA. At the same time, this model allows us to handle long DNA/RNA strands and to accumulate large statistics, which would be impossible from full first-principles calculations.

### Transport

The Hamiltonian of the total system, i.e., electrodes plus DNA/RNA molecule, has the form *H* = *H*_*S*_ + *H*_*L*_ + *H*_*R*_ + *H*_*cpl*_ where *H*_*S*_ is the molecule Hamiltonian, *H*_*L*/*R*_ is the Hamiltonian of the left/right electrode, and *H*_*cpl*_ is the coupling between the molecule and the electrodes. The Hamiltonian of the uncoupled DNA/RNA molecule is:

(6) $$H_{S}=\sum_{i\alpha }\varepsilon_{X_{i\alpha }}c_{i\alpha }^{†}c_{i\alpha }+\sum_{i\alpha ,j\beta }V_{X_{i\alpha }X_{j\beta }}c_{X_{i\alpha }}^{†}c_{X_{j\beta }}$$

where **ε**_*X*_*i*α__ and ***V***_*X*_*i*α_*X*_*j*β__ are the matrix elements of the nucleotides and the interaction between them, Equation (5) and *c*^†^_*X*_*i*α__ (*c*_*X*_*i*α__) are the electron creation (annihilation) operators on nucleotide *i* at fragment orbital α. The coupling between the electrodes and a particular nucleotide *X*_*i*_ of the DNA/RNA molecule is $H_{cpl}=\sum_{k,\mu }V_{X_{k}\mu }c_{X_{k}}^{†}c_{\mu }$, where ***V***_*X*_*k*_μ_ is the coupling between the electrode band and the MOs on the nucleotide. Finally, the electrodes are made of simple non-magnetic metals, such as Al and Au, can be treated on the level of a single-band TB model, $H_{L(R)}=\sum_{\mu }\varepsilon_{\mu }c_{\mu }^{†}c_{\mu }+\sum_{\mu ,\nu }t_{\mu \nu }c_{\mu }^{†}c_{\nu }$, where ε_μ_ is the on-site energy and *t*_μν_ is the hopping integral. However, treating the electrode explicitly can be avoided in this case, because the molecular levels are discrete, while the typical metal band is several electronvolts wide. Thus, we can treat the electrodes on the level of the wide band approximation (WBA). This implies that in the vicinity of the molecular level the density of states of the electrode is essentially constant and the interaction between the electrode band and the all MOs is the same. The main feature of the electrode is its chemical potential which is taken to be the electron work function (Singh-Miller and Marzari, 2009).

Given the Hamiltonian of the system of a molecule plus electrodes, we use the GF method to obtain the transverse current through base *k* as (Meir and Wingreen, 1992)

(7) $$I^{k}=\frac{2e}{h}\int dE\left(f_{L}\left(E\right)-f_{R}\left(E\right)\right)\text{ Tr}\left[\Gamma_{L}^{k}G\Gamma_{R}^{k}G^{†}\right]$$

where the semi-infinite left/right (*L*/*R*) electrodes are in equilibrium with chemical potentials μ_*L*_/μ_*R*_, *f*_*L*/*R*_ are the Fermi-Dirac distribution functions, Γ^*k*^_*L*/*R*_ are the escape rates to the electrodes when connected to base *k*, and *G* is the retarded GF of the DNA molecule connected to the electrodes. The calculation is performed in real space. To calculate the current we first diagonalize *H*_*S*_, Equation (6), to obtain the GF of the uncoupled DNA/RNA molecule *g*. Next we find the GF of the DNA/RNA coupled to the electrodes by solving the Dyson equation *G* = *g* + *g*Σ*G* where Σ = Σ_*L*_ + Σ_*R*_ is the self-energy due to the connection to the electrodes. Within WBA Σ_*L*/*R*_ and Γ_*L*/*R*_ = −2*Im*[Σ_*L*/*R*_] are constants independent of the energy. Since the DNA/RNA molecule is not chemically bonded to the electrodes, the contact is weak and we use Γ_*L*/*R*_ = 10^−3^eV in the calculations. The exact value of Γ_*L*/*R*_ is not very important because its effect is to scale the current, but it does not change the current distribution.

### Base calling

The identity of the base is decided based on the reading of the current through it. In the simplest procedure we use the current probability distribution functions (PDFs) for each base, *P*_1_ = *P*_*X*_ (*I*_*k*_), where *I*_*k*_ is the current through nucleotide *k* and *X* ∈ (*A*, *G*, *C*, *T*) for DNA and *X* ∈ (*A*′, *G*′, *C*′, *U*) for RNA. A base is assigned based on the maximum probability $\tilde{X}$_*k*_ = max_*X*_ *P*_*X*_ (*I*_*k*_) to measure this current through any of the bases. Due to the overlap in the current distributions, however, this base calling procedure is error prone.

Since the current spread is due to the influence of the neighbors, the currents through neighboring bases are not independent. In fact some features in the current PDF can be related to resonances in the transmission caused by interaction with the neighboring bases (Miroshnichenko et al., 2010; Alvarez et al., 2014). Therefore, including these current-current correlations can help alleviate the degeneracy in the current PDFs. This is the essence of a Bayesian improvement procedure we use to improve the accuracy of the base calls. The starting guess is the sequence obtained from single current PDFs, {$\tilde{X}$^(1)^_*k*_}. The information for the correlations between the currents through two, three, *etc*. bases, contained in the joint current PDFs, $P_{2}=P_{X_{1}X_{2}}(I_{k},I_{k+1}),P_{3}=P_{X_{1}X_{2}X_{3}}(I_{k},I_{k+1},I_{k+2})$, etc., is used to gradually improve on this guess. On each improvement step *n* the sequence guess from the previous step {$\tilde{X}$^(n − 1)^_*k*_} is used as a starting point. A new guess is obtained from the higher order distribution as the maximum of the joint PDF for a given site given that all other sites are fixed to those of the guess sequence, ${\tilde{X}}_{k}^{\left(n\right)}=\max_{X_{k}}P_{X_{k}|\{{\tilde{X}}^{\left(n-1\right)}\}}$. If for a given site the sequences are consistent, ${\tilde{X}}_{k}^{\left(n-1\right)}=={\tilde{X}}_{k}^{\left(n\right)}$, we assume this site to be certain and collapse the joint PDF such as it is one if ${\tilde{X}}_{k}^{\left(n\right)}$ is at site *k* and zero otherwise. After the certain sites are determined the sequence is recalculated using the modified joint PDF. The newly obtained sequence, $\left\{{\tilde{X}}_{k}^{\left(n\right)}\right\}$, is consistent with the order *n* PDF. Since the influence of the further neighbors is bound to be smaller, it is feasible to reduce the error rates below a desired threshold by construct higher order PDFs.

In order to test this procedure we use a two-step process. During the first step, we calibrate the method by constructing the joint current PDFs. This is achieved by collecting a large number of current readings for known DNA/RNA sequences (randomly generated). From these we gather together the currents through individual nucleotides, pairs, triples, etc. which are used to construct non-parametric joint PDFs. In principle, we can compute joint probability functions to any order with large enough statistics. In the second step, we use the obtained joint PDFs to compute the currents and call the bases in a test DNA/RNA sequence {*X*_*k*_}. As a metric of the success rate of such a sequencing procedure we define the *fidelity*:

(8) $$f=\frac{1}{N}\sum_{k=1}^{N}\left({\tilde{X}}_{k}==X_{k}\right)$$

where $\tilde{X}$_*k*_ is the guessed bases at position, *X*_*k*_ is the actual base, and the sum runs over all the *N* bases in the DNA/RNA sequence. Each correct identification $\tilde{X}$_*k*_ == *X*_*k*_ adds 1 to the sum and the measure is normalized to 1. The fidelity is complementary to the error rate.

## Results

### First-principles reduced model

**Figure 2:**
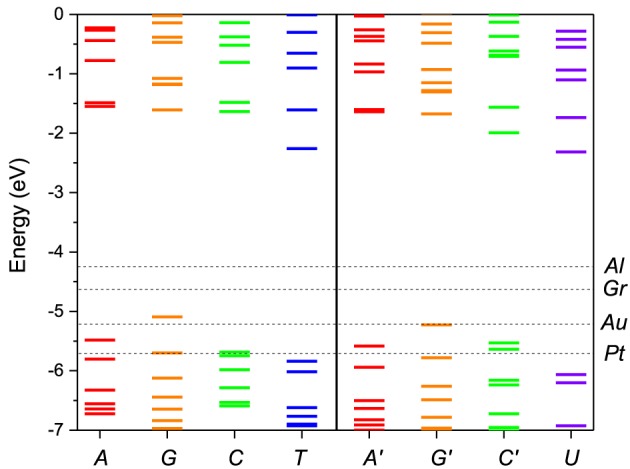
**Molecular energy levels of the of DNA (*****A*****,** ***G*****,** ***C*****,** ***T*****) and RNA (*****A*****′,** ***G*****′,** ***C*****′,** ***U*****) nucleotides calculated from first principles**. The Fermi levels of the metal electrodes, Al, Au, Pt, and Gr (graphene), are indicated by horizontal dashed lines. The purine bases are colored red/orange and the pyrimidine bases green/blue. The corresponding bases in DNA and RNA have the same color coding except for the T/U which are given in blue/violet to stress the difference in structure.

We performed first-principles calculations of the electronic structure of DNA and RNA single nucleotides and nucleotide pairs within ADF. Figure 2 shows the nucleotide energy levels for the four DNA nucleotides (*A*, *G*, *C*, *T*) and the corresponding RNA nucleotides (*A*′, *G*′, *C*′, *U*). Overall, we find that the nucleotide HOMO levels are in the range of −5.0 to −6.0 eV and the LUMO levels are approximately 4.0 eV higher in energy. This is consistent with previous DFT calculations (Roca-Sanjuán et al., 2006, 2008; Tsukamoto et al., 2009; Okamoto et al., 2012) and photoemission data (Lee et al., 2012). We also notice that the electronic structure of the corresponding DNA and RNA bases is very similar (*A*, *G* and *C*). The reason for that is that the backbone energy levels lie lower in energy and do not influence significantly the frontier orbitals. Therefore, the valence levels in all cases are determined mostly by the base itself. On the other hand the T levels are significantly different than those of U because in this case the modification is in the base itself.

In Figure 2 the nucleotide levels are also compared with the metal work functions. The relative position of the DNA/RNA orbitals with respect to the metal chemical potential determines which orbitals will participate in the transport. The first observation is that, although for some electrodes for most bias ranges (e.g., Al) the transport will remain in the tunneling regime, for most electrodes (e.g., Au, Pt) more than one energy level falls within the active window, which means that a multi-level model is essential to correctly describe the transport. The second observation is that the HOMO orbitals are in general closer to the active window and they will contribute more to the transport than the LUMO orbitals. For that reason we opt to include in the active window five MOs, four HOMO and one LUMO.

Next we construct the multi-level Hamiltonian of a polynucleotide molecule over an active energy region. We perform calculations of pairs of nucleotides, *XY*, using FMO theory where the MOs of the individual nucleotides serve as fragment orbitals. From these calculations we obtain the full onsite Hamiltonian matrix for each nucleotide and the hopping Hamiltonian matrix between two nucleotides. Furthermore, these large matrices are projected on the valence orbitals using projector operators to obtain small rank matrices. With this multi-band TB representation we can construct the Hamiltonian of any DNA/RNA chain.

**Figure 3:**
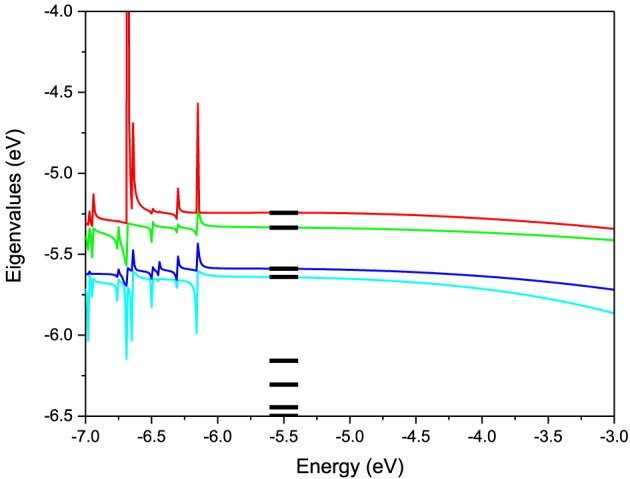
**Eigenvalues of the 2-level effective Hamiltonian of** ***AA*** **DNA pair as a function of energy**. The true molecular energy levels obtained from full first-principles calculations of the pair are given for comparison as bars.

To test this methodology we construct the effective Hamiltonian of a *AA* pair and compute its energy levels. As seen from Equation (4) the effective Hamiltonian is energy dependent. The levels of the pair constructed using the described methodology are shown as a function of energy in Figure 3 in comparison with the actual energy levels for the pair computed directly in ADF. For the energy values in the active energy region, the eigenvalues of effective Hamiltonian are smooth functions of the energy and agree very well with the eigenvalues of the total system. However, outside of this energy region singularities arise in the second term of ***H***_*eff*_(*E*) at the energy values equal to the eigenvalues of ***S***^−1^_*QQ*_***H***_*QQ*_. Based on these test calculations we find that the projection works extremely well in the desired active window. However, in order to use the effective Hamiltonian for a larger active window more valence levels have to be included in the model. Another observation is that the energy dependence of the Hamiltonian is very weak, which justifies a linearization of the model. Further on we use an energy independent Hamiltonian calculated from Equation (4) at an energy equal to the average energy of the valence orbitals.

### Statistical model calibration

We use the developed formalism to investigate the effect of the structural noise on the current distribution. First we calibrate the model to construct all joint PDFs. In order to collect sufficient statistics we generate DNA/RNA molecules consisting of 200 nucleotides where each of the bases appears randomly with the same probability. We calculate the current through each base of the 200 bases on 150 such sequences. In 30 of these sequences, poly(X) parts of varying length were introduced in order to train the procedure to recognize repeating sequences. From the resulting 30,000 observations we collect separately the currents through each base *X*, the pairs of currents through each base pair *XY*, and the triple of currents through each base triple *XYZ*. In each case a non-parametric PDF is constructed by making a fine histogram and interpolating it with a smooth function. The single PDF *P*_*X*_ (*I*_*k*_) has the meaning of the probability to measure current *I*_*k*_ through base *X*. There are 4 PDF for the current distribution through each nucleotide. The double PDF *P*_*XY*_ (*I*_*k*_, *I*_*k* + 1_) has the meaning of the probability to measure the currents *I*_*k*_ and *I*_*k* + 1_ through neighboring bases *X* and *Y*. There are 10 independent double PDFs. Similarly there are 40 independent triple PDFs for measuring triples of currents through three neighboring bases.

**Figure 4:**
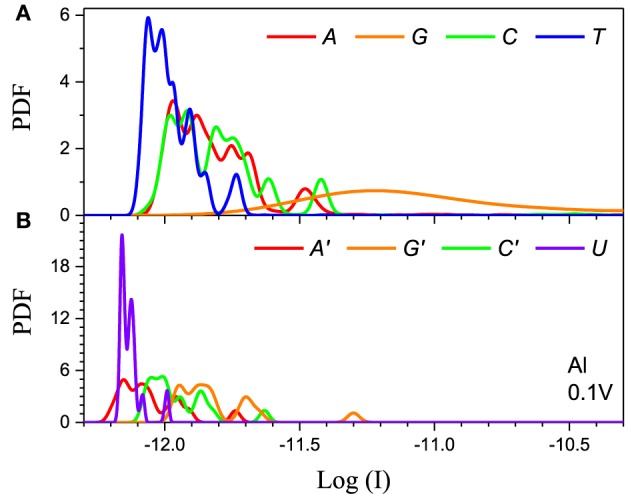
**Single current probability distribution function (not normalized) for all four bases in (A) DNA and (B) RNA within the 5-level model in the tunneling regime**. The calculation is performed with Al electrodes and 0.1 V applied bias. The color coding follows Figure 2.

**Figure 5:**
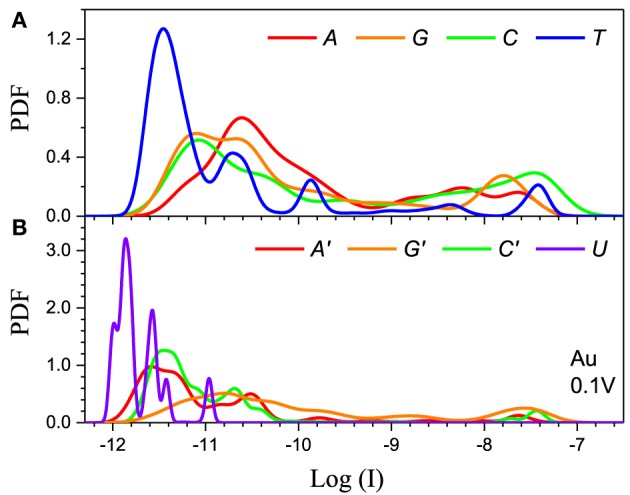
**Single current probability distribution function (not normalized) for all four bases in (A) DNA and (B) RNA within the 5-level model in the resonant regime**. The calculation is performed with Au electrodes and 0.1 V applied bias. The color coding follows Figure 2.

The method has to also be calibrated for each electrode. We consider two distinct cases: Al the chemical potential of which is well in the gap of the DNA/RNA and Au the chemical potential of which is aligned with the DNA/RNA HOMO levels (Figure 2). The resulting PDFs for the Al and Au electrodes for 0.1 V applied bias for the 5-level model are shown in (Figures 4, 5) respectively. The PDFs for the graphene (Gr) and Pt electrodes are given in the Supplementary Material. The most important observation is the obvious many orders of magnitude spread in the tunneling current in all cases and the corresponding large overlap of the current distributions, despite the lack of any environmental noise. This confirms that the structural noise indeed plays a major role in the error rates, which is unfortunate because this is an intrinsic property of the system and not the experimental setup.

The second feature is that the average current in the two cases is different by orders of magnitude, which implies that they clearly represent two distinct transport regimes. For small bias in the Al case the current is a pure tunneling current, while in the Au case molecular orbitals fall in the bias window and the current has a resonant character. Additionally, the current spread in the resonant regime is much larger leading to much stronger overlap between the current distributions. In the tunneling regime the order of the current distributions follows that of the HOMO levels (*G* > *A* ~ *C* > *T* in case of DNA and *G* > *C* > *A* > *U* in case of RNA), because the tunneling current depends exponentially on the barrier height (Figure 4). For the same reason the transport is dominated by the HOMO and the 5-level model PDFs are very similar to those obtained with the 1-level model (Alvarez et al., 2014).

In the resonant regime (Figure 5) the current spread is much larger and the PDF overlap much more pronounced. Each DNA/RNA nucleotide has *m* orbitals in the active window, thus a molecule with *n* nucleotides will have *nm* energy levels, many of which will be very close to the original *m* nucleotide levels due to hybridization (Miroshnichenko et al., 2010). If the nucleotide levels are within the bias window the neighboring levels will also give resonant tunneling contributions to the current on the same footing, which leads to the almost complete smearing out of the current because of the randomness of the chain. In contrast, in the tunneling regime the energy difference between the nucleotide and the satellite levels will translate into smaller satellite contributions to the current due to the exponential dependence of the current on the energy. In the resonant regime the 5-level model is essential to obtain correct results because lower lying orbitals give large contributions. For example, the 1-level model for Pt gives results similar to the tunneling regime (Alvarez et al., 2014), because the Pt electrode Fermi level is significantly below the HOMO. In the case of Pt the 5-level model gives a typical resonant PDF with large spread and overlap (see Supplementary Material). At the same time, the 5-level PDFs are less smooth than the PDFs from the 1-level model, with peaks corresponding to the contributions from the lower lying MOs.

Finally, the main difference between the DNA and RNA PDFs is the PDF of uracil. The *U* levels are lower than the rest of the nucleotides which causes the corresponding PDF to be sharper and have less overlap with the other nucleotides. This could lead to lower error rates for RNA sequencing compared to the equivalent DNA.

## Discussion

We have developed a methodology to calculate the transverse tunneling current through DNA/RNA molecules in the nanopore setup. The electronic structure of the molecule is on the level of a multi-level TB Hamiltonian derived from first-principles calculations by projecting the full molecule Hamiltonian on the energy window active in transport. This methodology allows us to investigate in both sufficient precision and sufficient detail the current spread due to the carrier dispersion along the chain of the molecule and the errors in base calling resulting from the overlapping of the current distributions.

### Error rates in base calling

First we address the main issue with DNA/RNA nanopore sequencing, namely the large error rates. We perform tests with both randomly generated DNA/RNA sequences and naturally occurring DNA sequences. The main difference between the two is that the natural sequences have a bias as certain bases appear more often than others in different organisms or in different parts of the genome, and the gene and regulatory sequences have constrained patterns of bases encoding genetic information. First, we consider a randomly generated DNA/RNA sequence composed of 200 base pairs (bps). We calculate the tunneling current through each base and using the current PDFs constructed in the calibration phase we call the bases. We repeat the procedure for four different electrodes Al, Gr, Au, and Pt at 0.1 V applied bias voltage on both DNA and RNA sequences. In addition, we compare the base calling procedure based on single PDFs with the Bayesian improvement procedure based on current-current correlations. As a measure of the success of the procedure we calculated the fidelity, Equation (8). A summary of the test results is given in Table 1.

**Table 1:** **Comparison of the calculated fidelity of the base calling for 200 bps randomly generated DNA/RNA sequences and naturally occurring DNA gene sequences (human insulin and BRCA1) with and without accounting for current correlations**.

	**Al**		**Gr**		**Au**		**Pt**	
	***P*_1_**	***P*_3_**	***P*_1_**	***P*_3_**	***P*_1_**	***P*_3_**	***P*_1_**	***P*_3_**
DNA (random, 200 bps)	0.645	*1.000*	0.655	*0.995*	0.400	*0.760*	0.455	*0.880*
RNA (random, 200 bps)	0.575	*1.000*	0.565	*1.000*	0.475	*0.980*	0.520	*0.925*
Insulin (324 bps)	0.682	*1.000*	0.679	*0.996*	0.417	*0.756*	0.438	*0.886*
BRCA1 (5592 bps)	0.658	*0.998*	0.657	*0.995*	0.508	*0.869*	0.388	*0.767*

The results are listed for several different electrodes at 0.1 V applied bias and low temperature.

The test shows that for the simple base calling the fidelity is indeed unacceptably low for any choice of the electrodes. In the resonant regime (Au, Pt) the fidelity is dismal, between 40 and 45% (55–60% error rate). In contrast to our previous results from the 1-level model (Alvarez et al., 2014), the fidelity in the case of Pt electrodes is comparable to that with Au electrodes due to the resonant contributions of the lower lying levels. In the tunneling regime (Al, Gr) the fidelity is significantly higher, around 65% (35% error rate), which underlines the importance of the choice of the electrodes. Nevertheless, this precision is not sufficient for applications.

Including the current correlations dramatically changes the picture. Including up to second order correlations essentially increases the fidelity of the base calling in the tunneling regime to 100%. Despite the large PDF overlaps in the resonant regime, we also observe a dramatic improvement in that case too with fidelities above 76%. The mechanism of this improvement is that certain features in the current distribution are explained by contributions from the neighboring bases. After these are stripped out the distributions are distinguishable. Thus, inclusions of correlations to a great extent resolves the problem with the accuracy of the nanopore sequencing. We also note that nanopore sequencing is equally well applicable to RNA. Actually, due to the greater difference in the electronic structure of the RNA bases the current distributions are more distinguishable and the error rates somewhat lower.

We also calculated the fidelity of the base calling for naturally occurring sequences (Table 1). First we consider insulin which is a hormone secreted in the pancreas and plays a major role in glucose metabolism. Insulin is the first protein for which the amino acid sequence was determined (Sanger and Tuppy, 1951). The other is the Breast Cancer 1 (BRCA1) susceptibility protein which is responsible for DNA repair and mutations of which have been correlated with the occurrence of breast cancer (Smith et al., 1996). The sequences were obtained from the European Nucleotide Archive, accession numbers AAA59179 and AAC37594 respectively^1^. The results shows that the procedure works very well in the case of natural sequences despite the fact that it has been calibrated with randomly generated sequences. The decrease in fidelity is almost unnoticeable in the tunneling regime (Al, Gr), but more pronounced in the case of the resonant tunneling regime (Au, Pt). This is an indication that bias should be taken into account in the training procedure in order to reduce the error rates.

### Error rates in sequence repeats

In addition to general errors consisting of misidentifying individual bases, a number of NGS technologies are prone to errors in differentiating repeating, XX… X, sequences embedded in the DNA chain (Metzker, 2010). These errors arise from the difficulty of separating multiple peaks of the same base and from the ambiguity of aligning the short reads generated by NGS technologies in the presence of regions of multiple repeating bases. Nanopore sequencing is capable of long read lengths and therefore not as susceptible to alignment errors. To test this premise, as a second test, we calculate the *partial* fidelity for correctly identifying short (triplets) and long (20 bases) repeating sequences appearing within otherwise random DNA sequence. In this case the sum in Equation (4) is not over all the bases but only over the repeated sequences. The results are presented in Table 2.

**Table 2:** **Comparison of the calculated partial fidelity of the base calling for poly(X)**_***n***_ **segments (*****n*** **= 3 or 20) inserted in a 200 bps random DNA sequence with and without accounting for current correlations**.

	**Al**		**Gr**		**Au**		**Pt**	
	***P*_1_**	***P*_3_**	***P*_1_**	***P*_3_**	***P*_1_**	***P*_3_**	***P*_1_**	***P*_3_**
AAA	0.778	*1.000*	0.778	*1.000*	0.555	*0.667*	0.333	*1.000*
GGG	0.556	*1.000*	0.889	*1.000*	0.444	*1.000*	0.778	*1.000*
TTT	0.667	*1.000*	0.833	*1.000*	0.333	*0.833*	0.160	*0.500*
CCC	0.333	*1.000*	0.500	*1.000*	0.222	*0.444*	0.333	*0.667*
A….A	0.928	*1.000*	0.969	*1.000*	0.878	*1.000*	0.033	*0.060*
G….G	1.000	*1.000*	0.682	*1.000*	0.091	*0.364*	0.955	*1.000*
T….T	1.000	*1.000*	0.968	*1.000*	0.968	*1.000*	0.781	*1.000*
C….C	0.955	*1.000*	0.929	*1.000*	0.071	*0.107*	0.000	*1.000*

The results are listed for several different electrodes at 0.1 V applied bias and low temperature.

Perhaps surprisingly we find that the transverse current sequencing is prone to these types of errors as well. For triplets using the simple base calling the probability for correctly identifying a *C* base in DNA with Al electrodes should be 65% (Table 1). However, when it appears in the *CCC* combination the probability is as low as 33%. Similarly the identification rate for the T base with Pt electrodes should be 46%, but it is as low as 16%. At the same time some cases show the opposite bias (e.g., *G* in triples with Pt electrodes). The reason for this behavior is that multiple identical bases hybridize strongly which each other creating levels which are very different from the original MOs. Fortunately, including current correlations essentially resolves this issue without special training. Again in the tunneling regime the fidelity approaches 100%. In the resonant regime the results are mixed. A larger bias window generally helps alleviate this problem.

For longer repeating sequences, however, we find that even including correlations does not improve significantly the fidelity. The reason is that for longer sequences the electronic structure of the nucleobase gradually changes from that of the molecular orbitals of an isolated molecule X to that of the electron band of a polymer of X, which are very different. Our calibrating procedure, based on limited statistics of randomly generated DNA, does not have enough long repeating sequences to influence the PDFs and therefore it misidentifies the reads. The results dramatically improve if during calibration we insert long repeating stretches. Exceptions are certain combinations of bases and electrodes (e.g., C with Au electrodes or A with Pt electrodes) for which the base is systematically misidentified because the currents from two of the bases in the poly(X) configuration are essentially degenerate for that electrode and applied bias (Alvarez et al., 2014).

Overall, our results indicate that a major source of error in nanopore sequencing via transverse current is the intrinsic noise coming from the disorder in the DNA/RNA chain itself. Fortunately, with the help of a statistical procedure taking into account the correlations between the currents through neighboring bases, these errors can in principle be reduced to levels comparable to those in NGS sequencing. This fact, in combination with the other advantages of nanopore sequencing could make this technology a viable alternative to NGS. Our results also indicate that, although alignment is not required for the nanopore sequencing procedure, repeating sequences pose a problem due to the high level of hybridization between the bases and the resulting modification of the electronic structure. Accounting for current correlations alleviates the problem for short base repeats where the influence of the neighbors on each of the bases is significant. However, for very long repeating sequences special calibration of the procedure is required to correctly call the bases.

### Conflict of interest statement

The authors declare that the research was conducted in the absence of any commercial or financial relationships that could be construed as a potential conflict of interest.

## Footnotes

^1^European Nucleotide Archive. Available online at: http://www.ebi.ac.uk/ena.

## Abbreviations

DNA: Deoxyribonucleic acid
RNA: Ribonucleic acid
A: Adenine
G: Guanine
C: Cytosine
T: Thymine
U: Uracil
NGS: Next-generation sequencing
bps: Base pairs
BRCA1: Breast Cancer 1
ADF: Amsterdam Density Functional
PBE: Perdew-Burke-Ernzerhof
TB: Tight-binding
WBA: Wide band approximation
TZP: Triple-zeta polarized
FMO: Fragment molecular orbitals
MO: Molecular orbitals
HOMO: Highest occupied molecular orbital
LUMO: Lowest unoccupied molecular orbital
GF: Green's function
PDF: Probability distribution function.

## References

[B1] Akeson M. Branton D. Kasianowicz J. J. Brandin E. Deamer D. W. (1999). Microsecond time-scale discrimination among polycytidylic acid, polyadenylic acid, and polyuridylic acid as homopolymers or as segments within single RNA molecules. Biophys. J. 77, 3227–3233. 10.1016/S0006-3495(99)77153-5 10585944 PMC1300593

[B2] Alvarez J. R. Skachkov D. Massey S. E. Lu J. Kalitsov A. Velev J. P. (2014). Intrinsic noise from neighboring bases in the DNA transverse tunneling current. Phys. Rev. Appl. 1:034001 10.1103/PhysRevApplied.1.034001

[B3] Apalkov V. Wang X. Chakraborty T. (2007). Physics aspects of charge migration through DNA, in Charge Migration in DNA, ed Chakraborty T. (Berlin; Heidelberg: Springer-Verlag), 77–119.

[B4] Ayub M. Bayley H. (2012). Individual RNA base recognition in immobilized oligonucleotides using a protein nanopore. Nano Lett. 12, 5637–5643. 10.1021/nl3027873 23043363 PMC3505278

[B5] Ayub M. Hardwick S. W. Luisi B. F. Bayley H. (2013). Nanopore-based identification of individual nucleotides for direct RNA sequencing. Nano Lett. 13, 6144. 10.1021/nl403469r 24171554 PMC3899427

[B6] Bayley H. (2006). Sequencing single molecules of DNA. Curr. Opin. Chem. Biol. 10, 628–637. 10.1016/j.cbpa.2006.10.040 17113816

[B7] Branton D. Deamer D. W. Marziali A. Bayley H. Benner S. A. Butler T.. (2008). The potential and challenges of nanopore sequencing. Nat. Biotechnol. 26, 1146–1153. 10.1038/nbt.1495 18846088 PMC2683588

[B8] Chang S. Huang S. He J. Liang F. Zhang P. Li S.. (2010). Electronic signatures of all four DNA nucleosides in a tunneling gap. Nano Lett. 10, 1070–1075. 10.1021/nl1001185 20141183 PMC2836180

[B9] Clarke J. Wu H.-C. Jayasinghe L. Patel A. Reid S. Bayley H. (2009). Continuous base identification for single-molecule nanopore DNA sequencing. Nat. Nanotechnol. 4, 265–270. 10.1038/nnano.2009.12 19350039

[B10] Cracknell J. A. Japrung D. Bayley H. (2013). Translocating kilobase RNA through the staphylococcal alpha-hemolysin nanopore. Nano Lett. 13, 2500–2505. 10.1021/nl400560r 23678965 PMC3712197

[B11] De Andrade P. C. P. Freire J. A. (2003). Effective Hamiltonians for the nonorthogonal basis set. J. Chem. Phys. 118, 6733–6740. 10.1063/1.1559485

[B12] De Andrade P. C. P. Freire J. A. (2004). Electron transfer in proteins: nonorthogonal projections onto donor-acceptor subspace of the Hilbert space. J. Chem. Phys. 120, 7811–7819. 10.1063/1.1690240 15267696

[B13] Dekker C. (2007). Solid-state nanopores. Nat. Nanotechnol. 2, 209–215. 10.1038/nnano.2007.27 18654264

[B14] Di Ventra M. (2013). Fast DNA sequencing by electrical means inches closer. Nanotechnology 24:342501. 10.1088/0957-4484/24/34/342501 23899780

[B15] Di Ventra M. Krems M. Wilson J. Pershin Y. V (2012). DNA characterization by transverse electrical current in a nanochannel, in Nanopore-Based Technology, Methods in Molecular Biology, ed Gracheva M. E. (Totowa, NJ: Humana Press), 149–163. 10.1007/978-1-61779-773-6_9 22528263

[B16] Fologea D. Gershow M. Ledden B. McNabb D. S. Golovchenko J. A. Li J. (2005). Detecting single stranded DNA with a solid state nanopore. Nano Lett. 5, 1905–1909. 10.1021/nl051199m 16218707 PMC2543124

[B17] Fonseca Guerra C. Snijders J. G. te Velde G. Baerends E. J. (1998). Towards an order- N DFT method. Theor. Chem. Acc. 99, 391–403. 10.1007/s00214005035323893735

[B18] Gershow M. Golovchenko J. A. (2007). Recapturing and trapping single molecules with a solid-state nanopore. Nat. Nanotechnol. 2, 775–779. 10.1038/nnano.2007.381 18654430 PMC3174059

[B19] Hawke L. G. D. Kalosakas G. Simserides C. (2010). Electronic parameters for charge transfer along DNA. Eur. Phys. J. E 32, 291. 10.1140/epje/i2010-10650-y 20680380

[B20] He J. Lin L. Zhang P. Spadola Q. Xi Z. Fu Q.. (2008). Transverse tunneling through DNA hydrogen bonded to an electrode. Nano Lett. 8, 2530–2534. 10.1021/nl801646y 18662039 PMC2575003

[B21] Heurich J. Cuevas J. C. Wenzel W. Schön G. (2002). Electrical transport through single-molecule junctions: from molecular orbitals to conduction channels. Phys. Rev. Lett. 88:256803. 10.1103/PhysRevLett.88.256803 12097112

[B22] Iguchi K. (2001). Semiconductivity and band gap of a double strand of DNA. J. Phys. Soc. Jpn. 70, 593–597. 10.1143/JPSJ.70.593

[B23] Ivanov A. P. Instuli E. McGilvery C. M. Baldwin G. McComb D. W. Albrecht T.. (2011). DNA tunneling detector embedded in a nanopore. Nano Lett. 11, 279–285. 10.1021/nl103873a 21133389 PMC3020087

[B24] Kasianowicz J. J. Brandin E. Branton D. Deamer D. W. (1996). Characterization of individual polynucleotide molecules using a membrane channel. Proc. Natl. Acad. Sci. U.S.A. 93, 13770–13773. 10.1073/pnas.93.24.13770 8943010 PMC19421

[B25] Kitaura K. Ikeo E. Asada T. Nakano T. Uebayasi M. (1999). Fragment molecular orbital method: an approximate computational method for large molecules. Chem. Phys. Lett. 313, 701–706. 10.1016/S0009-2614(99)00874-X

[B26] Korlach J. Turner S. W. (2012). Going beyond five bases in DNA sequencing. Curr. Opin. Struct. Biol. 22, 251–261. 10.1016/j.sbi.2012.04.002 22575758

[B27] Krems M. Zwolak M. Pershin Y. V. Di Ventra M. (2009). Effect of noise on DNA sequencing via transverse electronic transport. Biophys. J. 97, 1990–1996. 10.1016/j.bpj.2009.06.055 19804730 PMC2756358

[B28] Kurnikov I. V. Beratan D. N. (1996). Ab initio based effective Hamiltonians for long-range electron transfer: Hartree-Fock analysis. J. Chem. Phys. 105, 9561 10.1063/1.472788

[B29] Lagerqvist J. Zwolak M. Di Ventra M. (2006). Fast DNA Sequencing via transeverse electronic transport. Nano Lett. 6, 779–782. 10.1021/nl0601076 16608283 PMC2556950

[B30] Lagerqvist J. Zwolak M. Di Ventra M. (2007). Influence of the environment and probes on rapid DNA sequencing via transverse electronic transport. Biophys. J. 93, 2384–2390. 10.1529/biophysj.106.102269 17526560 PMC1965446

[B31] Lee Y. Lee H. Park S. Yi Y. (2012). Energy level alignment at the interfaces between typical electrodes and nucleobases: Al/adenine/indium-tin-oxide and Al/thymine/indium-tin-oxide. Appl. Phys. Lett. 101:233305 10.1063/1.4769438

[B32] Li J. Stein D. McMullan C. Branton D. Aziz M. J. Golovchenko J. A. (2001). Ion-beam sculpting at nanometre length scales. Nature 412, 166–169. 10.1038/35084037 11449268

[B33] Macia E. (2009). Charge transfer in DNA: effective Hamiltonian approaches. Z. Krist 224, 91–95. 10.1524/zkri.2009.112311235669

[B34] Maleki T. Mohammadi S. Ziaie B. (2009). A nanofluidic channel with embedded transverse nanoelectrodes. Nanotechnology 20:105302. 10.1088/0957-4484/20/10/105302 19417517

[B35] Meir Y. Wingreen N. S. (1992). Landauer formula for the current through an interacting electron region. Phys. Rev. Lett. 68, 2512–2515. 10.1103/PhysRevLett.68.2512 10045416

[B36] Metzker M. L. (2010). Sequencing technologies—the next generation. Nat. Rev. Genet. 11, 31–46. 10.1038/nrg2626 19997069

[B37] Miroshnichenko A. E. Flach S. Kivshar Y. S. (2010). Fano resonances in nanoscale structures. Rev. Mod. Phys. 82, 2257–2298. 10.1103/RevModPhys.82.2257

[B38] Ohshiro T. Matsubara K. Tsutsui M. Furuhashi M. Taniguchi M. Kawai T. (2012). Single-molecule electrical random resequencing of DNA and RNA. Sci. Rep. 2:501. 10.1038/srep00501 22787559 PMC3392642

[B39] Okamoto A. Maeda Y. Tsukamoto T. Ishikawa Y. Kurita N. (2012). A combined nonequilibrium Green's function/density-functional theory study of electrical conducting properties of artificial DNA duplexes. Comput. Mater. Sci. 53, 416–424. 10.1016/j.commatsci.2011.08.022

[B40] Purnell R. F. Mehta K. K. Schmidt J. J. (2008). Nucleotide identification and orientation discrimination of DNA homopolymers immobilized in a protein nanopore. Nano Lett. 8, 3029–3034. 10.1021/nl802312f 18698831

[B41] Roca-Sanjuán D. Merchán M. Serrano-Andrés L. Rubio M. (2008). Ab initio determination of the electron affinities of DNA and RNA nucleobases. J. Chem. Phys. 129, 095104. 10.1063/1.2958286 19044892

[B42] Roca-Sanjuán D. Rubio M. Merchán M. Serrano-Andrés L. (2006). Ab initio determination of the ionization potentials of DNA and RNA nucleobases. J. Chem. Phys. 125, 37–41. 10.1063/1.2336217 16965007

[B43] Roche S. (2003). Sequence dependent DNA-mediated conduction. Phys. Rev. Lett. 91:108101. 10.1103/PhysRevLett.91.108101 14525509

[B44] Sanger F. Nicklen S. (1977). DNA sequencing with chain-terminating inhibitors. Proc. Natl. Acad. Sci. U.S.A. 74, 5463–5467. 10.1073/pnas.74.12.5463 271968 PMC431765

[B45] Sanger F. Tuppy H. (1951). The amino-acid sequence in the phenylalanyl chain of insulin. 1. The identification of lower peptides from partial hydrolysates. Biophys. J. 49, 463. 14886310 10.1042/bj0490463 PMC1197535

[B46] Schadt E. E. Turner S. Kasarskis A. (2010). A window into third-generation sequencing. Hum. Mol. Genet. 19, 227–240. 10.1093/hmg/ddq416 20858600

[B47] Shapir E. Cohen H. Calzolari A. Cavazzoni C. Ryndyk D. A. Cuniberti G.. (2008). Electronic structure of single DNA molecules resolved by transverse scanning tunnelling spectroscopy. Nat. Mater. 7, 68–74. 10.1038/nmat2060 18037894

[B48] Shendure J. Ji H. (2008). Next-generation DNA sequencing. Nat. Biotechnol. 26, 1135–1145. 10.1038/nbt1486 18846087

[B49] Shih C. T. Roche S. Römer R. A. (2008). Point-mutation effects on charge-transport properties of the tumor-suppressor gene p53. Phys. Rev. Lett. 100, 1–4. 10.1103/PhysRevLett.100.018105 18232825

[B50] Singer A. Wanunu M. Morrison W. Kuhn H. Frank-Kamenetskii M. Meller A. (2010). Nanopore based sequence specific detection of duplex DNA for genomic profiling. Nano Lett. 10, 738–742. 10.1021/nl100058y 20088590 PMC2834191

[B51] Singh-Miller N. E. Marzari N. (2009). Surface energies, work functions, and surface relaxations of low-index metallic surfaces from first principles. Phys. Rev. B 80:235407 10.1103/physrevb.80.235407

[B52] Smith T. M. Lee M. K. Szabo C. I. Jerome N. McEuen M. Taylor M.. (1996). Complete genomic sequence and analysis of 117 kb of human DNA containing the gene BRCA1. Genome Res. 6:1029. 10.1101/gr.6.11.1029 8938427

[B53] Soriano M. Palacios J. J. (2014). Theory of projections with nonorthogonal basis sets: partitioning techniques and effective Hamiltonians. Phys. Rev. B 90:075128 10.1103/physrevb.90.075128

[B54] Stoddart D. Heron A. J. Mikhailova E. Maglia G. Bayley H. (2009). Single-nucleotide discrimination in immobilized DNA oligonucleotides with a biological nanopore. Proc. Natl. Acad. Sci. U.S.A. 106, 7702–7707. 10.1073/pnas.0901054106 19380741 PMC2683137

[B55] Storm A. J. Storm C. Chen J. Zandbergen H. Joanny J. F. Dekker C. (2005). Fast DNA translocation through a solid-state nanopore. Nano Lett. 5, 1193–1197. 10.1021/nl048030d 16178209

[B56] Te Velde G. Bickelhaupt F. M. Baerends E. J. Fonseca Guerra C. van Gisbergen S. J. A. Snijders J. G. (2001). Chemistry with ADF. J. Comput. Chem. 22, 931–967. 10.1002/jcc.1056

[B57] Timp W. Comer J. Aksimentiev A. (2012). DNA base-calling from a nanopore using a Viterbi algorithm. Biophys. J. 102, L37–L39. 10.1016/j.bpj.2012.04.009 22677395 PMC3353060

[B58] Tsukamoto T. Ishikawa Y. Sengoku Y. Kurita N. (2009). A combined DFT/Green's function study on electrical conductivity through DNA duplex between Au electrodes. Chem. Phys. Lett. 474, 362–365. 10.1016/j.cplett.2009.04.071 20160924 PMC2761638

[B59] Tsutsui M. Rahong S. Iizumi Y. Okazaki T. Taniguchi M. Kawai T. (2011). Single-molecule sensing electrode embedded in-plane nanopore. Sci. Rep. 1:46. 10.1038/srep00046 22355565 PMC3216533

[B60] Tsutsui M. Taniguchi M. Yokota K. Kawai T. (2010). Identifying single nucleotides by tunnelling current. Nat. Nanotechnol. 5, 286–290. 10.1038/nnano.2010.42 20305643

[B61] Wang Y. Yang Q. Wang Z. (2015). The evolution of nanopore sequencing. Front. Genet. 5:449. 10.3389/fgene.2014.00449 25610451 PMC4285804

[B62] Wanunu M. Sutin J. Meller A. (2009). DNA profiling using solid-state nanopores: detection of DNA binding molecules. Nano Lett. 9, 3498–3502. 10.1021/nl901691v 19585985 PMC2871189

[B63] Zwolak M. Di Ventra M. (2005). Electronic signature of DNA nucleotides via transverse transport. Nano Lett. 5, 421–424. 10.1021/nl048289w 15755087

[B64] Zwolak M. Di Ventra M. (2008). Colloquium: physical approaches to DNA sequencing and detection. Rev. Mod. Phys. 80, 141–165. 10.1103/RevModPhys.80.141

