# Supplementary material for: DNA/RNA transverse current sequencing: intrinsic structural noise from neighboring bases
Source: Front Genet. 2015 Jun 19;6:213. doi: 10.3389/fgene.2015.00213 (PMC4473640; doi:10.3389/fgene.2015.00213)
Supplement: Supplementary file 1 [file Presentation1.PDF]

Supplementary Material – DNA/RNA transverse current sequencing: Intrinsic structural noise from neighboring bases

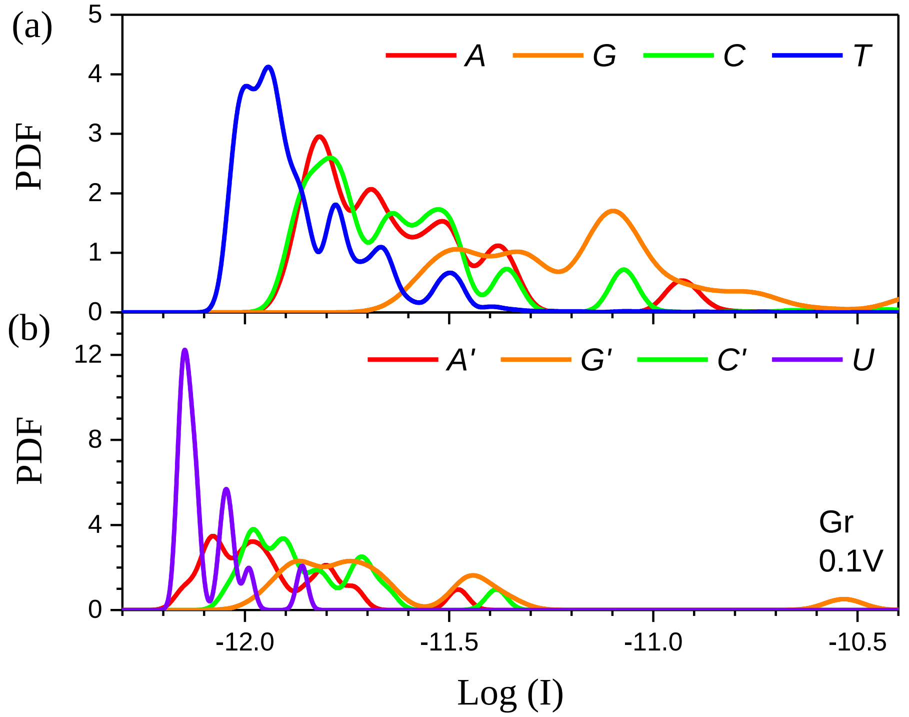

Figure S1. Single current probability distribution function (not normalized) for all four bases in (a) DNA and (b) RNA within the 5-level model in the tunneling regime. The calculation is performed with graphene (Gr) electrodes and 0.1V applied bias. The color coding follows Fig. 2.

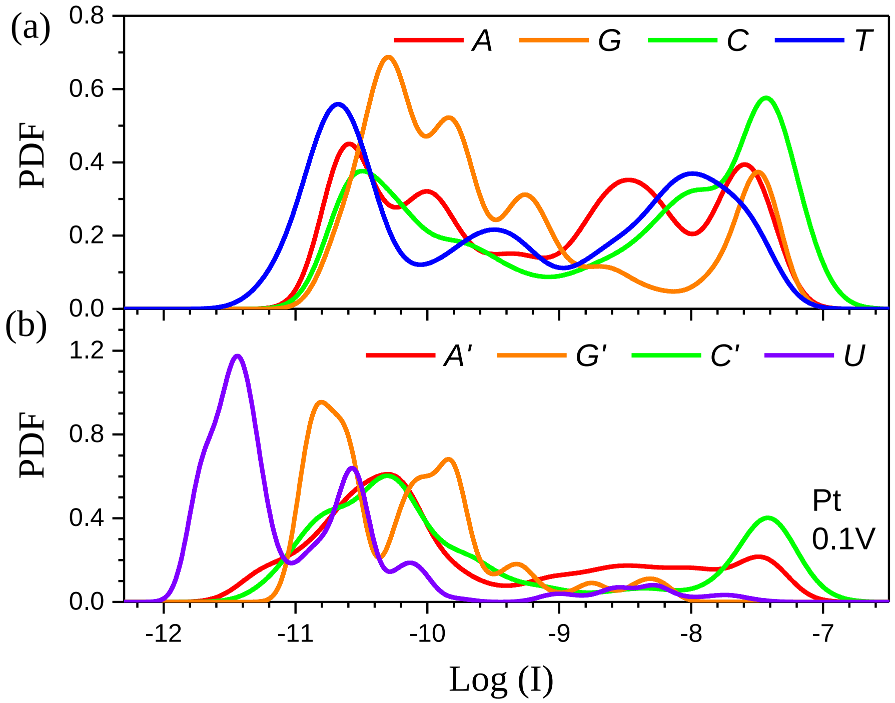

Figure S2. Single current probability distribution function (not normalized) for all four bases in (a) DNA and (b) RNA within the 5-level model in the resonant regime. The calculation is performed with Pt electrodes and 0.1V applied bias. The color coding follows Fig. 2.
